# Pulsatile liraglutide on a biased tirzepatide background: a hypothesis

**DOI:** 10.3389/fendo.2026.1922908

**Published:** 2026-09-10

**Authors:** Ivan Kopitovic

**Affiliations:** 1 Faculty of Medicine, University of Novi Sad, Novi Sad, Serbia; 2 Institute for Pulmonary Diseases of Vojvodina, Sremska Kamenica, Serbia

**Keywords:** biased agonism, GIPR, GLP-1R, incretin plateau, liraglutide, obesity, pulsatile dosing, tirzepatide

## Abstract

Tirzepatide (TZP) is a dual glucose-dependent insulinotropic polypeptide (GIP) and glucagon-like peptide-1 (GLP-1) receptor agonist that shows full agonism at the GIP receptor (GIPR) and reduced potency with G-protein-biased partial agonism at the GLP-1 receptor (GLP-1R) *in vitro*. Weight-loss response may attenuate during prolonged therapy, and some patients remain on lower doses after dose reduction for tolerability. Whether clinically relevant GLP-1R signalling capacity remains available for intermittent additional stimulation under these conditions is unknown. It is proposed that adding intermittent low-dose liraglutide to a stable low-dose TZP background, termed Receptor-Primed Episodic Agonism (RPEA), could be investigated as a way to re-engage GLP-1R signalling without increasing the TZP dose. Liraglutide has an elimination half-life of approximately 13 h, but residual systemic exposure may remain at 48 h and repeated q48h administration may accumulate; the interval therefore requires formal PK characterisation rather than being assumed to provide complete washout. RPEA is defined generally as intermittent short-acting full GLP-1R agonism on a stable TZP background; liraglutide, 0.6 mg, and q48–72h are illustrative candidates rather than defining features. An incremental response alone would not demonstrate priming; the defining test is a background-by-challenge interaction exceeding a prespecified additive null and not explained by a pharmacokinetic interaction. The concept rests on four explicitly unproven propositions: (i) residual GLP-1R signalling capacity during low-dose TZP; (ii) an incremental pharmacodynamic effect from episodic full GLP-1R agonism; (iii) less cumulative receptor adaptation with intermittent than continuous full-agonist exposure; and (iv) pharmacodynamic effects that may not track plasma concentration exactly. RPEA generates falsifiable preclinical and clinical questions but is not a recommendation for clinical use. No safety data are available for this off-label two-agent combination, and current prescribing information for both products does not recommend coadministration with another GLP-1 receptor agonist. The combination must not be used outside an ethics-approved clinical trial.

## 1 Introduction

The approval of tirzepatide (TZP), the first dual GIP/GLP-1 receptor agonist, marked a pharmacological inflection point in obesity medicine. In SURMOUNT-1, once-weekly TZP produced mean body-weight reductions of 16.0%–22.5% at 72 weeks in adults with obesity (1), and in the head-to-head SURMOUNT-5 trial, TZP produced greater mean weight reduction than semaglutide at 72 weeks (2). This observed difference has been discussed in relation to tirzepatide’s combined GIPR agonism and biased GLP-1R signalling, although the relative contribution of each receptor and signalling pathway to human weight loss and tolerability remains uncertain (3, 4).

A recognised limitation is attenuation of weight-loss velocity during maintenance therapy. It is important to distinguish deceleration of the weight-loss slope from a true plateau: SURMOUNT-1 showed continued but decelerating weight loss through 72 weeks rather than a fixed plateau (1), whereas maintenance and withdrawal studies demonstrate the need for continued therapy to maintain weight reduction (5). A clinically relevant subgroup comprises patients maintained on lower TZP doses following dose reduction for adverse effects or limited tolerability. The prevalence, mechanistic basis, and clinical trajectory of this subgroup have not been characterised prospectively and should not be inferred from the maintenance trial alone (5).

Weight reduction is the most consistently effective intervention for reducing obstructive sleep apnoea (OSA) severity, and SURMOUNT-OSA showed that TZP reduces the apnoea–hypopnoea index (AHI), hypoxic burden, C-reactive protein, and systolic blood pressure in adults with moderate-to-severe OSA and obesity, with the magnitude of improvement associated with weight loss (6–8). I state at the outset that this benefit is considered predominantly weight-mediated: the pharmacological hypothesis advanced here is obesity-general, while OSA is developed as a clinically motivating downstream application informed by my clinical practice rather than as a disease-specific pharmacological target.

Potential responses to an attenuated weight-loss response include reassessing adherence and secondary causes, escalating the dose where tolerated, switching therapy, or formally investigating adjunctive strategies. The hypothesis considered here addresses only the last possibility and does not imply that an untested combination should be used clinically. I therefore propose receptor-primed episodic agonism (RPEA) as a staged research concept that generates falsifiable receptor-level and pharmacodynamic questions (3, 4, 9).

### 1.1 How evidence is classified in this manuscript

Mechanistic language can convey more certainty than the underlying evidence supports. Rather than hedge each sentence individually, This manuscript utilises a single explicit convention, used consistently from this point forward and summarised comprehensively in **Table 1** (Section 9): claims in this manuscript fall into one of three evidence tiers: (1) established evidence, meaning directly demonstrated in the cited human or *in vitro* pharmacological studies; (2) pharmacological inference, meaning a reasonable extrapolation from established pharmacology to the untested RPEA scenario, not itself directly measured; and (3) novel hypothesis, meaning a claim specific to RPEA with no direct supporting data of any kind, animal or human. Readers should treat tier 3 claims, which include the central ‘priming’ premise, the 0.6-mg starter-dose premise, the kinetic β-arrestin-limitation premise, and the central pharmacodynamic-persistence premise, as the hypothesis under test, not as background fact. **Table 1** maps every major claim in the manuscript to its tier and to the section where it is developed.

**Table 1:** Evidence-tier classification of the core claims underlying RPEA.

Claim	Tier	Basis/section
TZP is a biased, partial GLP-1R agonist and a full GIPR agonist *in vitro*	1: Established	*In vitro* receptor pharmacology (3); Section 2.2
Liraglutide PK parameters (t½ ≈ 13 h, *C*_max_, *T*_max_)	1: Established	Published human PK (9); Section 2.4
GLP-1-induced tachyphylaxis of gastric emptying over hours (continuous vs. intermittent human infusion)	1: Established	(13, 14); Section 3.5
Hindbrain GLP-1R circuits mediating satiety vs. aversion are anatomically dissociable	1: Established (rodent circuit mapping; not this regimen)	(12); Section 3.7
Incremental GLP-1R signalling capacity may remain during lower-dose TZP therapy	2: Inference; not measured *in vivo*	Sections 2.3 and 3.3
Qualitative liraglutide exposure profile; residual exposure and accumulation require measurement	2: Inference from published PK	Section 4; **Table 3**; **Figure 1B**
Prior TZP exposure produces a background-by-challenge interaction exceeding a prespecified additive null and not explained by pharmacokinetic interaction	3: Novel hypothesis; defining discriminator	Sections 3.2–3.3 and 7 (P1)
A candidate agent, dose, and interval can be identified that reveal a reproducible RPEA interaction	3: Novel dose-/interval-finding hypothesis	Sections 1.1, 3.6, and 10
Intermittent and exposure-matched continuous full-agonist patterns differ in receptor adaptation	3: Novel hypothesis	Sections 3.5 and 7 (P3)
Central satiety persistence extends effective coverage beyond plasma clearance	3: Novel hypothesis (decoupled from interval choice)	Section 3.7
A demonstrated RPEA interaction has clinically meaningful relevance to weight-loss attenuation; OSA is a subsequent application	3: Novel hypothesis; clinical link unproven	Sections 5, 7, and 10

Tier 1 = directly demonstrated in cited studies. Tier 2 = pharmacological inference to the untested RPEA scenario. Tier 3 = novel hypothesis requiring direct testing.

A related terminological point concerns the level of generality at which RPEA itself should be defined. RPEA is best understood as the general hypothesis that intermittent, short-acting, full GLP-1 receptor agonism superimposed on a stable, lower-dose tirzepatide background can produce a background-dependent receptor-level or pharmacodynamic response that differs from the response expected under a prespecified additive null. An incremental response alone would not support priming. The specific parameters used elsewhere for illustration, liraglutide 0.6 mg and a q48–72h interval, are working examples chosen for feasibility and commercial availability, not defining features. The agent, dose, and interval are expected outputs of the preclinical and PK/PD stages in Section 10, not fixed inputs; a different short-acting agonist, dose, or interval that satisfies the same kinetic and safety logic would equally test RPEA.

## 2 Pharmacological background

### 2.1 GLP-1R signalling and desensitisation

The GLP-1 receptor is a class B1 G-protein-coupled receptor (GPCR) that, upon agonist binding, couples primarily to heterotrimeric Gs proteins, activates adenylyl cyclase, and generates cyclic adenosine monophosphate (cAMP), a canonical intracellular signal involved in insulin secretion and other GLP-1R-mediated physiological responses. Following activation, the receptor may be phosphorylated by G-protein-coupled receptor kinases, including GRK2 and GRK6, at serine and threonine residues within its intracellular domains. This phosphorylation promotes β-arrestin recruitment, which can contribute to G-protein uncoupling, receptor internalisation, and subsequent trafficking. Under sustained full-agonist exposure, these processes may reduce surface-receptor availability and attenuate signalling responsiveness (tier 1, established *in vitro* receptor pharmacology) (4).

Two temporal features are relevant to the present hypothesis. Receptor phosphorylation and β-arrestin recruitment can occur rapidly after agonist engagement, whereas internalisation, recycling, degradation, and restoration of signalling competence evolve over longer and system-dependent timescales. Dephosphorylation, β-arrestin dissociation, and receptor recycling may restore coupling competence after agonist exposure declines. However, the timing and functional importance of these processes during chronic GLP-1R agonist treatment in humans remain uncertain. Their application to RPEA is therefore a tier 2 pharmacological inference requiring direct validation rather than an established property of the proposed regimen (4).

### 2.2 Tirzepatide: imbalanced, biased dual agonism

TZP is an imbalanced dual GIP/GLP-1 receptor agonist *in vitro*. At GIPR, TZP behaves as a full agonist, whereas at GLP-1R, it shows reduced potency relative to native GLP-1 and preferential Gs/cAMP signalling with attenuated β-arrestin-2 and GRK2 recruitment (tier 1) (3, 4). This signalling profile has been proposed as a possible basis for differences in receptor trafficking and desensitisation relative to less-biased full GLP-1R agonists.

*In vitro* findings suggest that TZP may induce less GLP-1R internalisation than less-biased full agonists. However, the clinical relevance of this observation remains uncertain. Receptor surface expression, signalling capacity, trafficking behaviour, and tachyphylaxis during chronic TZP therapy have not been directly characterised in humans. Consequently, biased signalling provides a pharmacological rationale for investigation but does not establish preserved receptor availability or altered receptor adaptation in the proposed clinical setting.

### 2.3 Receptor availability during lower-dose TZP therapy

During lower-dose TZP therapy, continuing GIPR and biased GLP-1R agonism is expected. The extent of GLP-1R engagement, receptor reserve, surface expression, and capacity for an incremental response to another GLP-1R agonist have not been measured *in vivo* (3). It is therefore unknown whether lower TZP exposure leaves greater incremental GLP-1R signalling capacity than higher exposure or how large any such difference might be.

The RPEA hypothesis does not require a predefined pool of vacant receptors and does not assign numerical receptor-occupancy values. Instead, residual signalling capacity is treated as a tier 2 inference leading to a tier 3 testable proposition: whether an identical short-acting full-agonist challenge produces a different pharmacodynamic response on a stable TZP background than under the relevant control condition, exceeding a prespecified additive null and not explained by pharmacokinetic interaction.

### 2.4 Rationale for a shorter-acting GLP-1R agonist

The episodic component is represented by liraglutide because its elimination half-life is substantially shorter than that of weekly GLP-1R agonists. Published human pharmacokinetic data indicate a terminal half-life of approximately 13 h and a time to maximum concentration of approximately 8–12 h (9). These parameters predict a substantial post-peak decline but do not imply complete elimination or a biological off-state by 48 h. Residual systemic exposure may remain at that time, and repeated administration at 48-h intervals may produce accumulation. Both possibilities require direct pharmacokinetic characterisation.

Liraglutide is therefore presented as an illustrative candidate probe rather than a defining component of RPEA. The 0.6-mg dose is the labelled initiation dose, not an established effective dose for chronic weight management (9, 10). Similarly, q48h and q72h intervals are candidate schedules for formal investigation rather than pharmacokinetically validated intervals. Selection of the agent, exposure, and dosing interval must be guided by preclinical interaction assays, repeated-exposure studies, pharmacokinetic and pharmacodynamic modelling, and tolerability assessment. Neither plasma half-life alone nor an assumed receptor-recovery period is sufficient to determine an appropriate episodic schedule.

## 3 The RPEA concept

### 3.1 Three-layer signalling architecture

**Figure 1:**
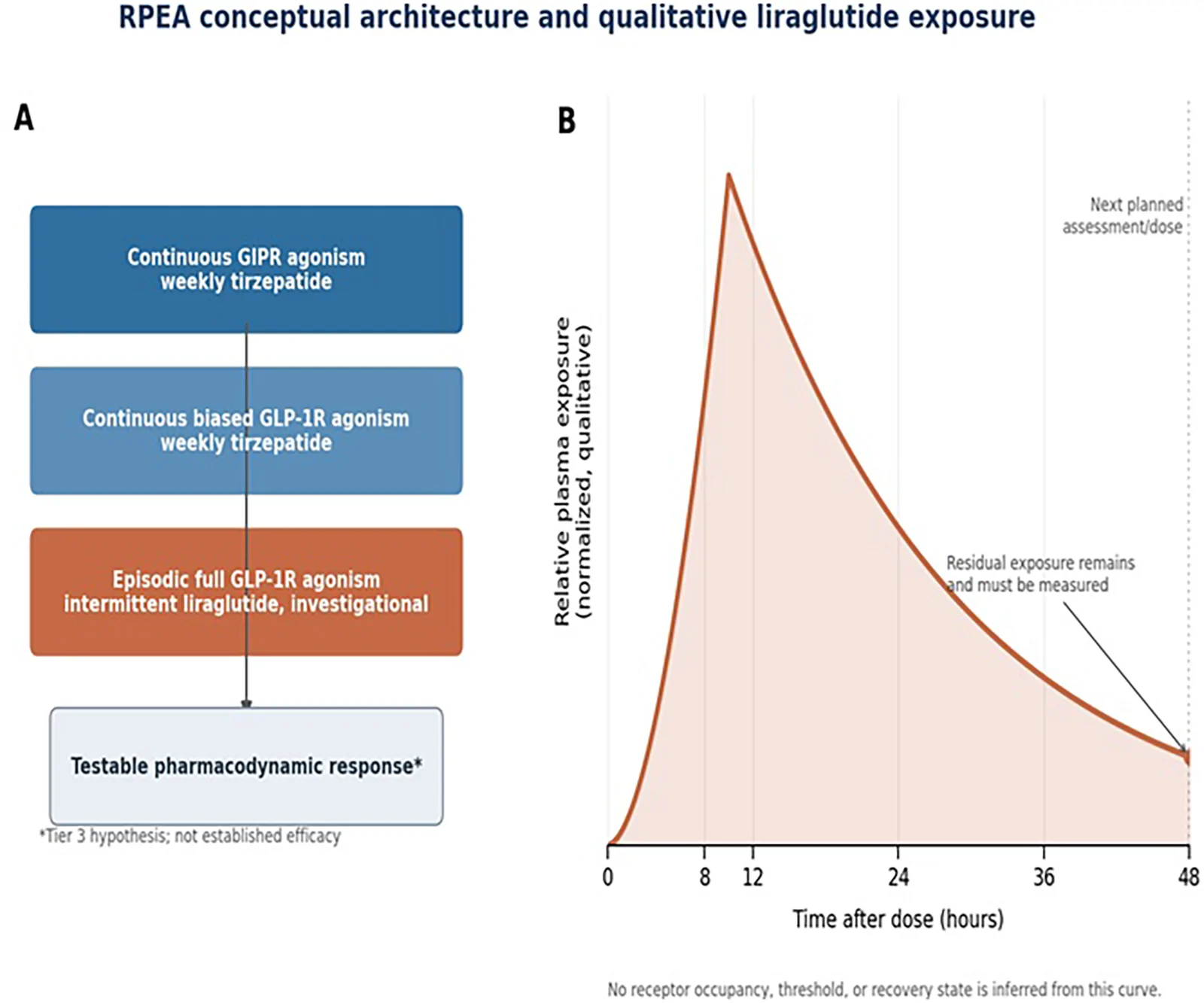
Conceptual schematic, not a fitted pharmacokinetic or pharmacodynamic model. **(A)** Proposed three-layer architecture: ongoing GIPR agonism and biased GLP-1R agonism from weekly tirzepatide, plus an investigational intermittent short-acting full GLP-1R challenge. Liraglutide is shown as one candidate probe. The defining tier 3 prediction is a background-by-challenge interaction exceeding a prespecified additive null and not explained by a pharmacokinetic interaction, not merely an incremental response. **(B)** Normalised qualitative single-dose liraglutide exposure based on the published approximate *T*_max_ and terminal half-life (9). The curve illustrates one candidate probe and does not define the RPEA dose or interval. Residual exposure remains at 48 h; repeated-dose accumulation, tissue exposure, receptor occupancy, β-arrestin recruitment, biological recovery, and optimal redosing are not estimated.

RPEA proposes three signalling layers operating on distinct timescales (**Table 2**). The specific agent (liraglutide), dose (0.6 mg), and interval (q48–72h) used throughout this architecture are illustrative working parameters for the general hypothesis defined in Section 1.1, not fixed requirements of the RPEA concept. The proposed three-layer signalling architecture is illustrated conceptually in **Figure 1A**.

**Table 2:** Proposed three-layer signalling architecture.

Signal layer	Driver	GLP-1R engagement	GIPR signal	Temporal pattern
GIPR continuous	TZP 5.0–7.5 mg	Concurrent GLP-1R agonism; magnitude not quantified	Ongoing agonism; clinical contribution not separable	Weekly exposure
GLP-1R biased	TZP 5.0–7.5 mg	Biased agonism established *in vitro*; *in vivo* engagement unknown	Concurrent	Weekly exposure
GLP-1R full intermittent	Investigational liraglutide	Full agonism; target engagement unknown	Concurrent TZP background	Candidate intermittent exposure

Receptor occupancy, relative GIPR contribution, β-arrestin engagement, and clinical efficacy are not quantified.

**Table 3:** Qualitative single-dose liraglutide exposure framework.

Time window	Relative liraglutide exposure	What is supported	What is not inferred	Evidence tier
After injection	Rising towards *T*_max_	*T*_max_ approximately 8–12 h (9)	Receptor occupancy or satiety threshold	Tier 1 PK; tier 3 PD
Near peak	Highest relative exposure	Peak occurs after delayed SC absorption (9)	β-Arrestin magnitude or circuit selectivity	Tier 1 PK; tier 3 mechanism
Post-peak	Declining	Terminal half-life approximately 13 h (9)	Complete receptor recovery	Tier 1 PK; tier 3 recovery
Hour 24	Residual exposure expected	Exposure has declined from peak	Near-zero occupancy	Tier 2
Hour 36	Further decline expected	Shorter persistence than weekly agonists	Biological off-state	Tier 2
Hour 48	Residual exposure may remain	Complete washout cannot be assumed	Optimal redosing time	Tier 2

The framework is illustrative and does not support inferences regarding absolute concentrations, receptor occupancy, β-arrestin-related thresholds, biological recovery, accumulation under repeated dosing, or an optimal dosing interval. Pharmacokinetic parameters are derived from Jacobsen et al. (9).

- Continuous GIPR layer (TZP 5.0–7.5 mg weekly): ongoing GIPR agonism from weekly tirzepatide. Its contribution to the net human pharmacodynamic response cannot be quantified separately from concurrent GLP-1R agonism (tier 1 for *in vitro* agonism; tier 2 for the clinical interpretation) (4).
- Continuous biased GLP-1R layer (TZP 5.0–7.5 mg weekly): ongoing biased GLP-1R agonism. Less internalisation than with less-biased full agonists is suggested by *in vitro* studies but has not been demonstrated during chronic human treatment (3, 4).
- Episodic full GLP-1R layer (investigational liraglutide): an intermittent full-agonist exposure superimposed on the TZP background. Its magnitude, duration, accumulation, receptor adaptation, and safety require direct measurement (9–11).

### 3.2 Reconciling receptor availability and receptor priming

Receptor availability and receptor priming are not treated here as established or competing structural states. Neither *in vivo* receptor occupancy nor a primed receptor state has been demonstrated in this context. Accordingly, priming is defined operationally as a prior TZP exposure state in which the incremental pharmacodynamic response to a standardised liraglutide challenge differs from the response observed without TZP background exposure. This definition provides a testable framework without presupposing receptor vacancy, altered receptor conformation, surface expression, trafficking, or sensitisation; any such mechanism would require direct receptor-level evidence.

An incremental appetite or weight response would not, by itself, demonstrate receptor priming. Potential alternative explanations include simple additivity, pharmacokinetic interaction, altered gastric emptying, regression to the mean, and expectancy effects or functional unblinding caused by adverse events. These explanations would require appropriate controls and, where feasible, mechanistic biomarkers.

Operational priming therefore remains a tier 3 hypothesis. It requires direct preclinical validation before consideration in any human dosing study. Establishing mechanistic priming would require not only a controlled pharmacodynamic response but also corroborating receptor-trafficking, occupancy, or signalling data.

To distinguish RPEA from simple additivity, the critical finding would be a prespecified background-by-challenge interaction in a factorial or randomised-crossover design: the incremental response to an identical short-acting full GLP-1R challenge would differ between stable TZP and control backgrounds relative to a prespecified additive null. This interaction would need to persist after paired PK measurements, baseline and period effects, altered gastric emptying, expectancy, and adverse-effect-related unblinding are addressed. Strong mechanistic support would additionally require concordant receptor-level evidence, such as altered GRK phosphorylation, β-arrestin recruitment, internalisation, recycling, or cAMP signalling in a TZP-conditioned system compared with the relevant single-agent conditions. If only an additive response is observed, or the apparent interaction disappears after PK adjustment, the priming component is rejected; the result may still support ordinary adjunctive agonism, but not RPEA as a distinctive mechanism.

The additive null must be specified *a priori*, separately for each experimental system and endpoint, rather than assumed generically. In preclinical assays, the additive null is the combined response predicted from the separately estimated TZP and challenge effects, relative to their common vehicle baseline and under matched exposure conditions, using the same prespecified readout. The exact response scale and additive model must be defined *a priori* for each assay and endpoint. In human PK/PD studies, the null is the combined pharmacodynamic response predicted from the TZP-alone and challenge-alone conditions under an additive model prespecified for the endpoint, response scale, and study design. Candidate approaches may include effect additivity, dose additivity, or another justified reference model. A statistically significant deviation from this null establishes only that the combined response is non-additive; it does not by itself demonstrate receptor priming or any other specific mechanism, since a non-additive interaction could instead reflect a pharmacokinetic interaction, an unmodelled physiological confound, or a statistical artefact arising from misspecification of the null model. Confirmation of priming as defined in this manuscript therefore additionally requires the receptor-level evidence specified above, not the interaction result in isolation.

The value of this framework lies not only in how the experiments are sequenced but also in what a fully positive result would support. If the background-by-challenge interaction, exposure-pattern-dependent receptor adaptation, and a durable weight effect are confirmed sequentially, RPEA would provide evidence supporting a receptor-level mechanism in which residual GLP-1R responsiveness during an attenuated weight-loss response differs between intermittent and exposure-matched continuous full-agonist conditions. Establishing causality or mediation directly would require the receptor-level and mechanistic biomarker evidence specified in Section 10, not the interaction result alone. Pure additivity predicts no such background-by-challenge interaction, while a reproducible exposure-pattern-dependent difference in receptor adaptation would provide an additional, mechanistically distinct line of support. Such a result would constitute substantially stronger evidence than an expected additive pharmacodynamic pattern, rather than merely attaching a mechanistic label to it. The staged design in Section 10 is intended to ensure that this interpretation is earned through convergent evidence rather than assumed at any single stage.

### 3.3 Mechanism 1: residual signalling capacity and operational priming (tier 2 premise → tier 3 hypothesis)

During stable lower-dose TZP therapy, the central premise is that an intermittent liraglutide challenge could produce an incremental GLP-1R-mediated pharmacodynamic response. The premise does not require or claim a measured pool of vacant receptors. The defining test is whether prior TZP exposure modifies the response to an identical challenge relative to the prespecified additive null, after pharmacokinetic interaction, expectancy, and other competing explanations have been addressed. A qualifying interaction would establish background dependence, not its receptor-level cause (3, 4).

### 3.4 Mechanism 2: differential signalling elicited by a full-agonist challenge (tier 1 receptor pharmacology → tier 2/3 application)

GPCRs can adopt multiple conformational states, and agonists may differ in efficacy and signalling bias (tier 1). Whether a liraglutide challenge on a TZP background produces a qualitatively or quantitatively distinct central GLP-1R signal is unknown (tier 3) (3, 4, 12). The manuscript therefore treats any putative central activation threshold as a testable conceptual construct rather than a measured biological constant.

### 3.5 Mechanism 3: exposure-pattern-dependent receptor adaptation (tier 3)

β-Arrestin engagement is not eliminated by intermittent liraglutide and cannot be inferred from plasma concentration alone. The narrower claim here is that intermittent exposure may produce a different pattern of receptor adaptation than continuous full-agonist exposure. This remains a tier 3 hypothesis, and no numerical occupancy threshold or fixed recovery window is assigned. The supporting human evidence is indirect. Nauck et al. demonstrated rapid tachyphylaxis of GLP-1-induced deceleration of gastric emptying during continuous infusion, and Umapathysivam et al. found that intermittent stimulation with a recovery interval preserved gastric-emptying effects that were attenuated by prolonged stimulation (13, 14). Extrapolation from short-term gastric-emptying physiology to receptor-density regulation or sustained weight loss over weeks to months is a tier 2–3 inference. Repeated-pulse receptor assays are therefore required before human evaluation.

This gap warrants explicit narrowing rather than a bridging assumption. Gastric-emptying tachyphylaxis and receptor-density regulation over weeks to months are not established to represent the same biological process, and this manuscript does not assume they do. The claim is restricted to a sequential test: first, whether intermittent and exposure-matched continuous full-agonist conditions differ in receptor adaptation; and second, only if such a difference is demonstrated, whether it corresponds to a durable change in appetite or weight trajectory. If repeated-pulse stability is not observed at the receptor level, the proposed adaptation mechanism is unsupported. If receptor-level stability is observed without a corresponding long-term weight effect, it cannot explain attenuation of weight loss.

### 3.6 The initiation dose as an illustrative candidate challenge (tier 3)

The 0.6-mg dose is the labelled initiation dose, whereas 3.0 mg once daily is the recommended adult dose for chronic weight management (10). It is retained only as one conservative candidate challenge for preclinical exposure matching and, if justified, formal dose-finding. Neither this dose nor q48h/q72h administration defines RPEA. Failure to demonstrate a meaningful interaction at the starting dose of 0.6 mg would reject that candidate challenge, not the general framework; failure to identify any background-by-challenge interaction across justified agents, exposures, and intervals would reject RPEA itself. Empirical dose escalation outside a formally approved programme is not supported.

### 3.7 Mechanism 4: pharmacodynamic persistence relative to measured exposure (tier 3)

Plasma concentration and pharmacodynamic effect need not have identical time courses, but the duration of central appetite effects after a liraglutide challenge on a TZP background is unknown (12). Recent circuit mapping shows anatomically dissociable hindbrain GLP-1R populations involved in satiety and aversion in rodents (tier 1 for that finding) (12), but this does not establish preferential circuit engagement by a low dose or by the proposed combination. Central persistence is retained only as a tier 3 question to be tested by paired concentration and pharmacodynamic measurements.

The dosing interval is not justified by an assumed period of central persistence or complete drug washout. Candidate intervals should instead be selected after preclinical and early human PK/PD characterisation, including residual exposure and accumulation under repeated dosing. If pharmacodynamic effects track plasma exposure closely, the persistence hypothesis is rejected.

## 4 Qualitative pharmacokinetic framework for the liraglutide pulse

No population pharmacokinetic model, individual-level dataset, receptor-occupancy model, or simulation underlies this framework. Published pharmacokinetic parameters for liraglutide include a terminal half-life of approximately 13 h and a time to maximum concentration of approximately 8 to 12 h (9). A simple terminal-phase calculation suggests that residual systemic exposure may remain 48 h after administration and that repeated administration at 48-h intervals may produce some accumulation. These observations are qualitative and do not establish an optimal dosing interval.

Total plasma liraglutide concentration cannot be reliably translated into GLP-1 receptor occupancy without validated information on unbound concentrations at relevant receptor compartments, tissue distribution, receptor affinity under physiological conditions, receptor reserve, and the influence of concurrent TZP exposure. **Figure 1B** therefore presents only an illustrative, normalised single-dose exposure profile. It does not estimate absolute concentrations, receptor occupancy, β-arrestin-related thresholds, biological recovery, or an optimal dosing interval.

Three implications follow. First, shorter persistence than weekly GLP-1R agonists does not mean complete elimination at 48 h. Second, any recovery interval relevant to receptor trafficking must be measured rather than inferred from plasma half-life. Third, repeated-dose accumulation and the background TZP concentration must be incorporated into any formal PK/PD model. These considerations make PK characterisation and repeated-pulse receptor assays prerequisites for schedule selection.

A q48h schedule is retained only as one candidate interval for investigation, not as a kinetically optimal schedule. q72h and other intervals should be compared after formal modelling and measurement of single-dose and repeated-dose exposure, pharmacodynamic response, tolerability, and receptor adaptation. **Figure 1B** is intentionally qualitative and should not be used to infer a dosing recommendation.

## 5 Weight-mediated relevance to obesity-related OSA

None of the four proposed mechanisms is OSA-specific; any relevance to obesity-related OSA is expected to be weight-mediated. Consistent with the obesity-general framing in Sections 1.1 and 10, OSA is treated here only as a downstream, weight-contingent application (stage 5), not as an initial test population, and this section is kept brief for that reason.

OSA affects roughly 30%–40% of adults with obesity and up to 70%–80% of those with severe obesity, and SURMOUNT-OSA established TZP as disease-modifying in this population, reducing AHI, hypoxic burden, and systolic blood pressure in proportion to weight loss (6–8). Patients whose weight-loss response attenuates at lower TZP doses may not reach the weight threshold associated with meaningful AHI improvement (5, 6). Bedtime administration may be evaluated as a prespecified factor in future OSA-specific studies, but no respiratory safety advantage has been established.

If RPEA produces a clinically meaningful weight effect in the general obesity-attenuation population (stage 4), a subsequent, weight-contingent OSA study should test whether renewed weight loss corresponds to AHI and hypoxic-burden improvement, alongside PAP adherence and cardiometabolic markers, consistent with SURMOUNT-OSA data (6). OSA benefit is not expected in the absence of further weight loss and is not a defining prediction of the receptor mechanism (P6, Section 7).

## 6 Comparison with alternative approaches

- TZP dose re-escalation. Not viable in patients dose-reduced for tolerability and non-selective: it raises GIPR and GLP-1R signalling proportionally rather than selectively augmenting GLP-1R (1, 3).
- Addition of semaglutide. Semaglutide has a substantially longer half-life than liraglutide and would not provide a short exposure interval (4, 9). However, comparative receptor displacement, β-arrestin recruitment, and autonomic effects of add-on semaglutide on a TZP background have not been measured. This alternative is not recommended clinically and would require the same preclinical and regulatory scrutiny.
- GLP-1R positive allosteric modulators (PAMs). PAMs such as LSN3160440 enhance G-protein signalling without displacing orthosteric ligand or recruiting β-arrestin, a theoretically ideal complement to TZP, but none is clinically approved. RPEA is a pharmacologically reasoned approximation using approved agents, accepting orthosteric competition in exchange for immediate investigability.
- Triple agonists (retatrutide). Agents with additional glucagon-receptor agonism represent a different pharmacological strategy. Such agents remain investigational and are not currently approved, and their comparative efficacy, tolerability, and effects on obesity-related OSA in patients unable to tolerate TZP re-escalation cannot be inferred and would require direct study.

## 7 Testable predictions and falsification criteria

RPEA generates six falsifiable predictions:

- P1. Discriminating interaction: In a factorial or randomised-crossover design, the incremental response to an identical short-acting full GLP-1R challenge differs between stable TZP and control backgrounds beyond a prespecified additive null. The interaction must persist after paired PK sampling, baseline and period effects, gastric-emptying differences, expectancy, and adverse-effect-related unblinding are addressed. An incremental response alone, or an interaction that disappears after PK adjustment, does not support priming.
- P2. PK/PD relationship: The time course of appetite-related effects may differ from the measured liraglutide concentration–time curve. Persistence beyond a prespecified concentration range would support PK/PD hysteresis; close tracking would reject the persistence hypothesis.
- P3. Exposure-pattern-dependent receptor adaptation: Intermittent and exposure-matched continuous full-agonist conditions produce different receptor-signalling or trafficking profiles across repeated challenges. If no difference is observed, the proposed adaptation advantage is rejected; if a receptor difference is observed, its relevance to long-term weight outcomes remains a separate hypothesis.
- P4. Tolerability and safety: Adverse events, heart rate, blood pressure, glycaemia, gastrointestinal symptoms, gallbladder events, pancreatic safety signals, renal events, and sleep-related outcomes are characterised prospectively. No tolerability advantage is assumed.
- P5. Weight-loss relevance: Only after the defining interaction and receptor-adaptation prediction are supported should a trial test whether participants with obesity and a protocol-defined attenuation of weight-loss response show clinically meaningful re-engagement. Renewed weight loss without the prespecified interaction would be interpreted as additivity or another mechanism rather than evidence for RPEA.
- P6. Subsequent weight-contingent OSA application: After the core interaction and a clinically meaningful weight effect are established, a separate OSA study may test whether additional weight loss is accompanied by AHI and hypoxic-burden improvement. OSA benefit is not a defining prediction of the receptor mechanism.

## 8 Proposed investigational protocol elements (not clinical guidance)

Safety statement: read before this section. There are no safety data for coadministration of tirzepatide and liraglutide. Plausibly additive or interacting risks require formal nonclinical assessment and independent safety oversight. Current prescribing information for both Zepbound and Saxenda does not recommend coadministration with another GLP-1 receptor agonist (10, 11). Everything in Section 8 is an outline for ethics and regulatory discussion, not a treatment recommendation. This combination must not be used outside an ethics-approved clinical trial.

The elements below identify questions that a future protocol would need to resolve. They are not a dosing regimen and should not be used for self-directed or off-trial treatment.

### 8.1 Investigational protocol parameters (for use only within an ethics-approved trial)

- Background population: Adults on a stable, tolerated lower TZP dose with a prospectively defined attenuation of weight loss, after adherence, secondary causes, and alternative approved management strategies have been assessed.
- Investigational component: Liraglutide exposure levels and candidate intervals should be selected from preclinical receptor assays and formal PK/PD modelling. A 0.6-mg challenge and q48h or q72h intervals are hypotheses for dose-finding, not recommended schedules.
- Timing: Morning versus evening administration may be evaluated as a prespecified factor in OSA studies. No respiratory or tolerability benefit of bedtime dosing is assumed.
- Interval selection: This is determined from measured single-dose and repeated-dose exposure, accumulation, pharmacodynamic duration, receptor-adaptation markers, and tolerability rather than from half-life alone.

### 8.2 Proposed trial monitoring procedures

These are candidate monitoring domains for ethics-committee and regulatory review, not a monitoring plan for off-trial use. Early studies should include serial PK sampling, prespecified appetite and satiety measures selected from the preceding modelling, heart rate and blood pressure, systematic gastrointestinal grading, glycaemia, renal and hepatobiliary safety, pancreatic safety signals, body composition, and complete adverse-event recording. In OSA-specific studies, objective sleep testing, hypoxic burden, PAP-adherence data, and validated symptom questionnaires should be included at protocol-defined timepoints.

### 8.3 Illustrative safety-governance elements

Consistent with the hypothesis-only framing of this paper, the following are offered as examples of the oversight any first-in-human evaluation would require, not as a recommended protocol or as elements the author is qualified to finalise.

- Independent oversight: Data and Safety Monitoring Board (DSMB) with a charter for scheduled and *ad hoc* safety review
- Sentinel dosing: staggered, sentinel-first initiation with a mandatory observation interval between the first participants before wider enrolment
- Prespecified no-go/stopping criteria: explicit thresholds; for example, defined rates or severity of gastrointestinal, autonomic, or vestibular adverse events, protocol-defined hypoglycaemia, or any pancreatitis signal, that trigger dose-hold, de-escalation, or study termination
- Exclusion and re-initiation rules: predefined exclusion criteria and a re-titration rule for participants who miss doses, given that repeated re-initiation following missed-dose intervals may recreate gastrointestinal intolerance

These items would need to be specified and approved by investigators with formal clinical-development and trial-safety expertise.

## 9 Limitations and evidence gaps

**Table 1** gives the complete, systematic classification of every major claim used in this manuscript, tagged by evidence tier and linked to the section where it is developed. The bullet points that follow add detail specific to each gap rather than repeating the tier classification already visible in the table.

- No occupancy estimates are presented: *In vivo* GLP-1R occupancy, receptor reserve, and surface expression under low-dose TZP are unmeasured. Total plasma liraglutide concentration cannot be translated reliably into receptor occupancy in this setting (Sections 2.3 and 4).
- No prospective combination data: No study has evaluated any GLP-1R agonist added to a TZP background; the combination is entirely unstudied clinically and for safety.
- Distinctiveness from additivity is unproven (tier 3): An incremental response is insufficient. RPEA requires a reproducible background-by-challenge interaction beyond a prespecified additive null, persisting after PK adjustment and control of competing explanations. If only additivity is observed, receptor priming is rejected.
- The bridge to weight-loss attenuation is unproven (tiers 2–3): Short-term gastric-emptying tachyphylaxis and receptor-trafficking observations do not establish a cause of long-term attenuation (13, 14). Receptor-pattern, short-term pharmacodynamic, and durable weight outcomes must be tested sequentially rather than treated as one biological process.
- Plateau is multifactorial: Metabolic adaptation, neurohormonal counter-regulation, and behaviour contribute; RPEA addresses only receptor-level and acute central components.
- The OSA pathway is weight-mediated: The chain from receptor pharmacology to AHI passes through weight loss and requires independent prospective validation (Section 5).

## 10 Call for formal investigation

Although both component drugs are individually approved for weight management, their combination is not an established or recommended treatment. Current U.S. prescribing information for Zepbound and Saxenda does not recommend coadministration with another GLP-1 receptor agonist (10, 11). RPEA can therefore be considered only within a formally approved, ethics-approved, and regulatorily-compliant investigational framework. Human dosing is not the first step.

### 10.1 Stage 0: preclinical/translational validation

Before any human dosing study, the defining RPEA discriminator should be tested in reductionist systems using factorial background-by-challenge conditions and an explicit additive null, so that ordinary co-agonism is not relabelled as priming. The following elements are proposed:

- Interaction assays: Quantify cAMP signalling, receptor phosphorylation, β-arrestin recruitment, internalisation, recycling, and surface expression across TZP/control backgrounds and episodic full-agonist/vehicle challenges. The critical result is a reproducible background-by-challenge interaction, not merely a larger combined response.
- Exposure and target engagement: Measure liraglutide and TZP concentrations and, where technically feasible, receptor target engagement or internalisation in suitable experimental systems. These data should replace qualitative exposure illustrations before schedule selection.
- Repeated-pulse stability: Test whether repeated challenges preserve signalling capacity or produce cumulative desensitisation across clinically relevant exposure patterns, including residual drug and accumulation.

Only if these experiments support the defining interaction, rather than merely an additive combined response, should the human studies below proceed.

### 10.2 Stage 1: PK/PD interaction characterisation in TZP-maintained patients

If preclinical interaction criteria are met, an early human factorial or randomised-crossover study should estimate the background-by-challenge interaction on validated appetite, gastric-emptying, metabolic, and tolerability measures, with paired TZP and probe exposure measurements. The sample size should estimate the interaction and its variability, not clinical efficacy.

### 10.3 Stage 2: dose and interval finding

Candidate short-acting full GLP-1R agonists, exposures, and intervals, potentially including liraglutide, a 0.6-mg challenge, and q48h or q72h schedules, should be compared only after formal exposure and interaction modelling. Selection criteria should include interaction reproducibility, residual concentration, accumulation, pharmacodynamic duration, receptor-adaptation findings, and tolerability. No dose or interval is privileged *a priori*.

### 10.4 Stage 3: longitudinal receptor-adaptation study

A longitudinal study should assess whether pharmacodynamic effects and safety remain stable across repeated challenges. Duration and sampling frequency should be justified from preceding PK/PD data rather than fixed in advance.

### 10.5 Stage 4: randomised proof-of-concept trial in obesity with characterised weight-loss attenuation

Reflecting the obesity-general framing adopted throughout this manuscript (Sections 1.1 and 5), the initial randomised proof-of-concept trial should enrol adults with obesity and a rigorously characterised, prospectively documented attenuation of weight loss on stable lower-dose TZP, rather than obesity-related OSA specifically. This population provides the most direct test of the central RPEA hypothesis, since the primary and secondary outcomes (P1, P3, and P5 in Section 7) are receptor- and weight-related, not respiratory. Dose, interval, eligibility criteria, endpoints, and sample size should be determined from pilot estimates of treatment effect and variance within a prespecified statistical analysis plan. Obesity-related OSA should be considered as a subsequent, weight-mediated application (stage 5 below) once the core RPEA mechanism has been established in the general obesity population with characterised weight-loss attenuation, rather than as the first proof-of-concept setting.

### 10.6 Stage 5: weight-contingent extension to obesity-related OSA

Only if stages 0–4 establish the defining interaction and a clinically meaningful effect on weight-loss trajectory in the general obesity-attenuation population should a dedicated trial in obesity-related OSA test the weight-contingent hypotheses in Section 5 and P6. This sequencing retains OSA as a clinically important downstream application of an obesity-general hypothesis rather than the initial mechanistic test.

## 11 Conclusion

The RPEA hypothesis proposes investigating intermittent short-acting full GLP-1R agonism on a stable lower-dose TZP background. Its defining prediction is not merely an incremental response, but a background-by-challenge interaction exceeding a prespecified additive null and not explained by pharmacokinetic or procedural factors. If only an additive response is observed, or if an apparent interaction disappears after pharmacokinetic adjustment, the priming component is rejected. Liraglutide, a 0.6-mg challenge, and q48h or q72h administration are illustrative candidates rather than defining features. Short-term gastric-emptying and receptor-trafficking findings motivate exposure-pattern experiments but do not establish a mechanism for long-term attenuation of weight loss. Clinical relevance must therefore be evaluated sequentially, first in people with obesity and rigorously characterised attenuation of weight-loss response, and only subsequently in obesity-related OSA as a predominantly weight-mediated application.

RPEA is a framework for investigation, not a recommendation for clinical adoption. The proposed combination remains clinically unvalidated and should be evaluated only within an appropriately approved investigational programme. If the defining interaction, exposure-pattern-dependent receptor adaptation, and a durable effect on weight trajectory are confirmed sequentially, the framework would provide evidence supporting a receptor-level mechanism by which intermittent, rather than continuous, full GLP-1R agonism may re-engage weight loss in patients with attenuated response. A qualifying interaction alone would not establish mechanistic causality or mediation, which would require concordant receptor-level and mechanistic biomarker evidence. Pure additivity predicts no qualifying background-by-challenge interaction and would therefore not support RPEA as a distinctive pharmacological framework. The staged agenda in Section 10 begins with preclinical and translational validation rather than human dosing and provides the falsifiable tests required before further development.

## Data Availability

The original contributions presented in the study are included in the article/supplementary material. Further inquiries can be directed to the corresponding author.
